# A Clinical Trial Evaluating the Efficacy of Deep Learning-Based Facial Recognition for Patient Identification in Diverse Hospital Settings

**DOI:** 10.3390/bioengineering11040384

**Published:** 2024-04-15

**Authors:** Ayako Sadahide, Hideki Itoh, Ken Moritou, Hirofumi Kameyama, Ryoya Oda, Hitoshi Tabuchi, Yoshiaki Kiuchi

**Affiliations:** 1 Department of Ophthalmology and Visual Science, Graduate School of Biomedical Sciences, Hiroshima University, Hiroshima 734-8551, Japan; sadahideayako@hiroshima-u.ac.jp (A.S.); ykiuchi@hiroshima-u.ac.jp (Y.K.); 2 Division of Patient Safety, Hiroshima University Hospital, Hiroshima 734-8551, Japan; 3 GLORY Ltd., Himeji 670-8567, Japan; moritou.ken@mail.glory.co.jp (K.M.); kameyama@mail.glory.co.jp (H.K.); 4 Graduate School of Advanced Science and Engineering, Hiroshima University, Hiroshima 739-8526, Japan; ryoya-oda@hiroshima-u.ac.jp; 5 Department of Ophthalmology, Tsukazaki Hospital, Himeji 671-1227, Japan; h.tabuchi@tsukazaki-eye.net; 6 Department of Technology and Design Thinking for Medicine, Hiroshima University Graduate School, Hiroshima 734-8551, Japan

**Keywords:** facial recognition, deep learning, patient identification

## Abstract

Background: Facial recognition systems utilizing deep learning techniques can improve the accuracy of facial recognition technology. However, it remains unclear whether these systems should be available for patient identification in a hospital setting. Methods: We evaluated a facial recognition system using deep learning and the built-in camera of an iPad to identify patients. We tested the system under different conditions to assess its authentication scores (AS) and determine its efficacy. Our evaluation included 100 patients in four postures: sitting, supine, and lateral positions, with and without masks, and under nighttime sleeping conditions. Results: Our results show that the unmasked certification rate of 99.7% was significantly higher than the masked rate of 90.8% (*p* < 0.0001). In addition, we found that the authentication rate exceeded 99% even during nighttime sleeping. Furthermore, the facial recognition system was safe and acceptable for patient identification within a hospital environment. Even for patients wearing masks, we achieved a 100% success rate for authentication regardless of illumination if they were sitting with their eyes open. Conclusions: This is the first systematical study to evaluate facial recognition among hospitalized patients under different situations. The facial recognition system using deep learning for patient identification shows promising results, proving its safety and acceptability, especially in hospital settings where accurate patient identification is crucial.

## 1. Introduction

Facial recognition systems are a kind of technology that identifies individuals by recognizing individual facial images [1]. They have been employed in settings other than hospitals [2,3], e.g., in security cameras [4], examinations for immigration [5], and entrance and exit control systems in offices, libraries [6], and stations [7]. These systems have proven to be beneficial in each field. Significantly, facial recognition systems that utilize deep learning techniques dramatically improve the accuracy of facial recognition technology. The correct response rate for facial recognition is approaching almost 100% [8]. Widely used mobile applications can now identify facial images with a 99.7% valid response rate and a recognition speed of 0.5 s [9]. It is well known that wearing masks reduces face recognition accuracy, as with iPhones and other devices [10,11]. However, according to a recent study of masked face recognition [12], authentication is possible regardless of the type or position of the mask [13]. 

Facial recognition is now expected to play an important role in future healthcare systems. The adoption of facial recognition systems in hospitals is expected to reduce human error and prevent patient misidentification. Wrong-patient errors occur in all steps of diagnosis and treatment in hospitals, highlighting the need to improve the accuracy of patient identification. In the United States, approximately 400,000 hospitalized patients experience some preventable medical error each year [14]. The ratio of patient error to serious medical errors is not low: 4% and 14% in and outside the operating room, respectively [15]. In this regard, the Joint Commission considers patient identification one of the goals for patient safety [14]. For example, there have been cases where medical staff have called out a patient’s name and the wrong person has walked into the exam or procedure room, resulting in mistaken patients and medication errors [16]. The Japanese Ministry of Health, Labor and Welfare reported 144 cases of medical accidents due to patient errors between January 2019 and December 2021, most often in hospitalized patients, and the most common place where patient errors have occurred is in hospital rooms [17].

Moreover, patient recognition in cases involving the loss of consciousness or in-hospital patient management overnight is another unsolved issue. A study using a surveillance system in a dementia ward found that 10-30% of patients moved during the night [18]. The common wristband recognition system used while a patient is sleeping may wake the patient, and the nurses may hesitate to use it [19,20]. In a multicenter study of 712 U.S. hospitals, identification by wristband resulted in 67,289 (2.7%) errors, 49.5% of which were lost ID bands [21]—wristbands do not work if they come off or are lost [22]. Therefore, other methods of correctly recognizing patients in hospitals are required.

In facial recognition systems, recognition rates decrease under conditions such as certain lighting or angles, wearing accessories such as masks or glasses, and low resolution [23]. Advances in AI technology have improved recognition rates under these conditions [24,25]. However, special usage situations not envisioned in these studies are possible in hospitals. For facial recognition in hospitals, it is necessary to consider conditions such as lying on a bed or dimmed lighting. The illuminance required for face recognition is 200 lux or higher. Silverstein showed that when imaging the same face in different light levels from 60-285 lux, the process is less accurate in lower light and only captures consistent face data if the ambient light is sufficient [8]. Munn and Stephan pointed out that facial recognition performance declines when a person is lying down because of the physical change in their facial expression by gravity [26]. Therefore, verifying the facial recognition system is necessary in hospitals for various patient postures and for a low-light environment during nighttime sleeping where the illuminance is 60 lux or lower. 

In addition to the current methods mentioned above for patient recognition, an appropriate facial recognition system could ensure patient safety. Therefore, this study evaluated whether a facial recognition system supported by deep learning could help correctly recognize patients in different hospital-related situations, including mask-wearing and nighttime sleeping (closed eyes, low illumination, and supine or both lateral positions). We also used an iPad to take facial images, because this a mobile device that could be available in various situations in hospitals and because the resolution of the built-in camera in the iPad is higher than that of fixed cameras.

## 2. Materials and Methods

### 2.1. Participants

This study enrolled 100 patients (66 males; mean height of 162 ± 9 cm; mean weight of 61 ± 10 kg; mean body mass index of 23 ± 3) who underwent surgery at the Department of Ophthalmology, Hiroshima University Hospital. The patients were 20 or older (mean age of 68 ± 13 years), and 46 patients wore glasses. All patients agreed to participate in this study and provided written informed consent. The Institutional Review Board of Hiroshima University approved this study (No E2021-2693). This study adhered to the tenets of the Declaration of Helsinki. Patients under 20 years of age and those who could not follow instructions to open or close their eyes were excluded; all other patients were included in this study.

### 2.2. Image Acquisition Technique

On the day of admission, a single ophthalmologist took facial photographs of all the patients who consented to participate in this study in an examination room without windows. We used an iPad Air, 4th generation (Apple Inc., Cupertino, CA, USA) with a photo resolution of 3024 × 4032 pixels and an illuminance meter (HIOKI FT3424, Nagano, Japan) to take the photos. The facial images had a 120 mm × 80 mm frame.

**Figure 1:**
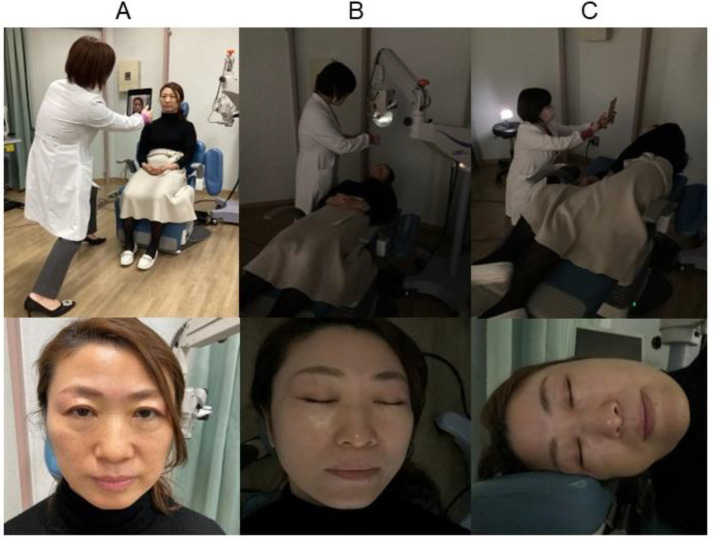
(**A**) Standard photography; (**B**) supine position with low illumination; (**C**) left lateral position with low illumination.

A total of 1900 facial photographs were taken, including control photographs and 18 patterns of photographs of 100 patients. Among 18 patterns, 16 patterns were combinations of 3 conditions (eyes, position, illumination) with or without mask, and 2 patterns were right or left lateral position without mask. The control photographs were taken under the normal condition (open eyes, sitting, sufficient illumination). We fully recognized the faces in all photos that were detected and extracted. For each patient, we obtained facial photographs in the control standard condition (open eyes, sitting) and the adverse condition. The control standard condition consisted of no mask, sitting position, open eyes, and sufficient illumination (Figure 1A). The adverse condition included wearing a mask, supine position, closed eyes, and low illumination. We first examined 16 conditions, which consisted of every combination of two patterns of mask-wearing (with or without a mask), four body positions (sitting or supine), two eye conditions (open or closed), and two illumination conditions (sufficient or low illumination). In addition, we evaluated 2 patterns, including the right and left positions, under the assumption of nighttime sleeping conditions. In the images obtained with the patient wearing a mask, the upper edge covered the nasal wings and the apex of the nose, and the lower edge covered the chin. The sitting position means the patient was sitting with their upper body at a 90-degree angle, while in the supine position, the patient was lying on their back. The right lateral position means that the patient was lying on their side with their right side down. The left lateral position means they were lying on their side with their left side down.

In the images obtained under sufficient illumination in the examination room, the mean illuminance with lighting was 656 ± 74 lux in the sitting position and 536 ± 60 lux in the supine position. The mean illuminance was between 3 and 4 lux in the images obtained with low illumination. When in a supine position with low illumination (Figure 1B), the light from the ophthalmic operation microscope (OMS-90, TOPCON, Tokyo, JAPAN) was adjusted by covering it with gauze to illuminate the front of the face at a brightness of 3-4 lux. And when the patient was in the side lateral position, the photography light (Ulanzi VL49, Guangdong, China) was directed from the front (Figure 1C).

### 2.3. Authentication Score of the Facial Recognition System with Deep Learning

We used a 1:1 authentication method to identify the correct person against a reference image and calculated the authentication score (AS) using the latest facial recognition system with deep learning. We performed facial recognition using the ISP-417 facial recognition development kit (Glory, Hyogo, Japan) and Glory’s V5-5 engine-generated score values. This system consists of three steps: facial image extraction, feature extraction, and distance calculation. In the facial image extraction step, the face to be authenticated was detected and extracted from a photo. In the next step, approximately 2000 features representing individual facial differences, such as the eyes, nose, mouth, and forehead, were extracted using convolutional neural networks. Glory trained the neural network as supervised learning models from a dataset of over 10 million. AS was expected to increase if additional learning could be carried out under adverse conditions (low illumination, eyes closed, supine position). In the third step, we calculated the AS based on the distance between the features extracted from the two face images, ranging from 0 to 1. In order to obtain the AS, we calculated the cosine similarity (*CS*) between a vector of the reference image (v*_a_*) and a vector (v*_b_*) of the targeted image, as below:

$$CS=\frac{v_{a}\cdot v_{b}}{\left|\left|v_{a}\left|\left|\;\right|\right|v_{b}\right|\right|}$$

AS = 0.5 × *CS* + 0.5

A higher AS indicates that the two facial images belong to the same person. We confirmed that the reproducibility of the AS values calculated multiple times on the same pair of images was 100%.

### 2.4. Thresholds Based on the False Rejection and False Acceptance Rates (FRRs and FARs)

A low FAR indicates more excellent safety in patient recognition. In contrast, a low FRR shows more accurate identification of the appropriate patients. We calculated the FRR and FAR with and without masks.

To assess whether a patient would be correctly or wrongly identified compared to the targeted person, we defined a threshold based on the FAR, which reflected the rate at which the system identified a wrong individual as the targeted person as the minimum AS. We set AS thresholds for masked and unmasked subjects because the minimum AS with a false acceptance rate (FAR) of 0% differs between masked and unmasked patients. Our system automatically recognizes whether a person is wearing a mask or not.

### 2.5. Statistics

Statistical analysis was conducted using JMP PRO software (version 16; SAS Institute Inc., Cary, NC, USA). Welch’s *t*-test was used to compare the mean AS of a correct and wrong patient and the frequency of successful matches for each situation. All statistical tests were two-sided, and statistical significance was set at a *p*-value of < 0.05.

## 3. Results

**Figure 2:**
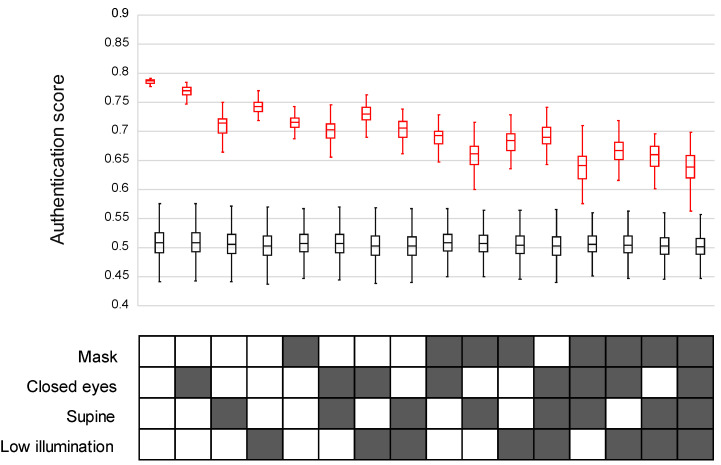
Authentication scores (AS) in 16 patterns depending on the combination of masking, eyes, body position, and illumination. The AS is shown in the upper half, and the 16 patterns are in the lower half. The AS of intentionally wrong patients was significantly lower than that of correct patients for each situation (*p* < 0.0001). In the upper panel, red boxes = scores for correct patients, and black boxes = scores for wrong patients. In the lower panel, solid boxes = conditions of mask, closed eyes, supine, and low illumination.

We built the authentication score (AS) (0 (worst)–1 (best)) to evaluate the internal conviction degree to identify the correct person against a reference image. As shown in Table 1, which summarized all the authentication scores, the AS was significantly lower under the adverse conditions (closed eyes, low illumination, supine position, or mask-wearing) than the standard conditions (open eyes, sufficient illumination, sitting position, and mask-free) (0.767 ± 0.012, 0.741 ± 0.012, 0.710 ± 0.020, 0.714 ± 0.015 vs. 0.785 ± 0.004; n = 100 in each condition; *p* < 0.0001), and the AS declined as these factors increased (Figure 2). In the facial recognition of a wrong patient, the AS was 0.50 ± 0.02 in all situations. This was significantly lower than the AS of the correct patient for each situation (*p* < 0.0001).

**Figure 3:**
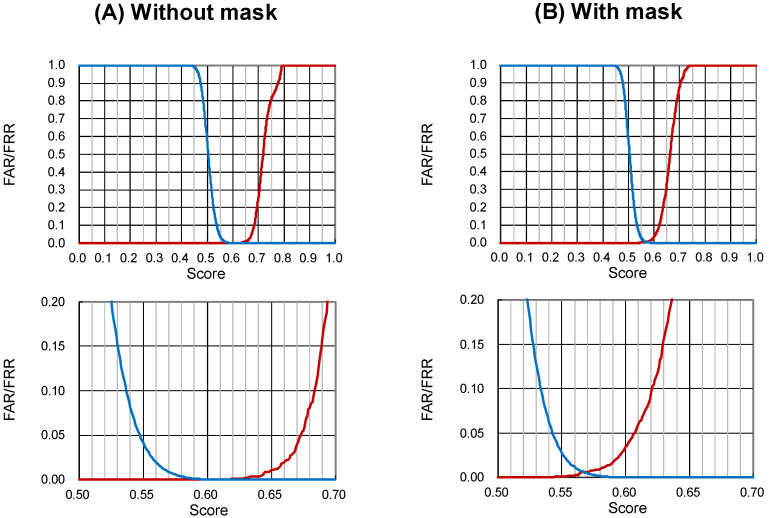
False rejection rate (FRR) and false acceptance rate (FAR) under conditions (**A**) without and (**B**) with a mask. The upper panels show the total range, and the lower panels extend the crossing ranges between the FRR and the FAR. The threshold is the maximum score of wrong matches among all 158,400 values. The thresholds without and with masks were 0.642 and 0.620, respectively. FRR = red lines, FAR = blue lines.

By setting the lowest AS value (0.642 without mask, 0.620 with mask), which is the lowest value for which the false acceptance rate (FAR) is 0%, as the lower threshold of the match decision, we calculated the correct response rate (Figure 3). 

**Figure 4:**
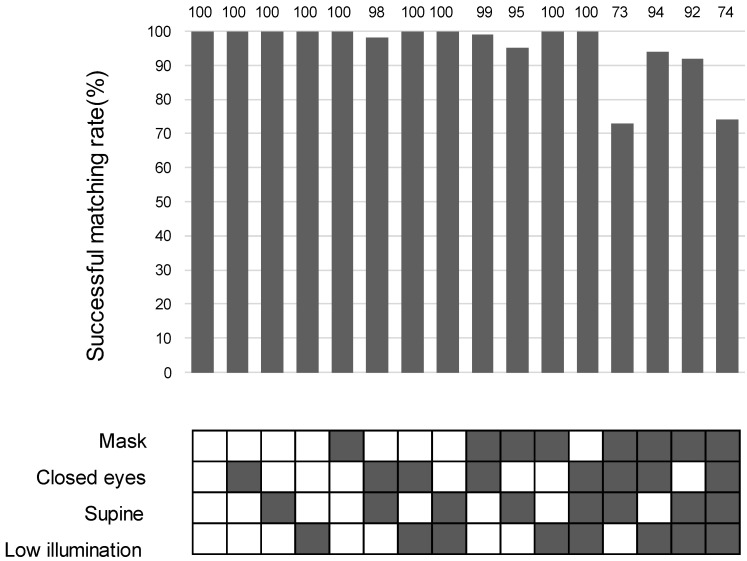
Frequency of successful matches in 16 combinations of mask-wearing, eye closure, body position, and illumination. The rate of successful matches can be indicated as [(1-FRR) × 100 (%)]. The frequency of successful matches is shown in the upper part, and the 16 patterns are in the lower part. In the lower panel, solid boxes = conditions with mask, closed eyes, supine, sufficient or low illumination. The exact numbers are provided in the top row of the matching rate bar chart.

Our results show that the unmasked certification rate of 99.7% (798/800) was significantly higher than the masked rate of 90.8% (727/800). Under the adverse conditions (closed eyes, supine position, low illumination) except the standard condition (open eyes and sitting under sufficient illumination), the mean (SD) success rate of authentication was 99.7% (698/700) without a mask, and 89.5% (627/700) with a mask, which was significantly worse under mask-wearing conditions. (*p* < 0.0001, Figure 4).

The AS values under the nighttime sleeping condition (no mask, low illumination, closed eyes, and any of the three conditions (supine, right side lateral, and left side lateral) were 0.691 for the supine position, 0.701 for the right-side lateral position, and 0.702 for the left-side lateral position.

**Figure 5:**
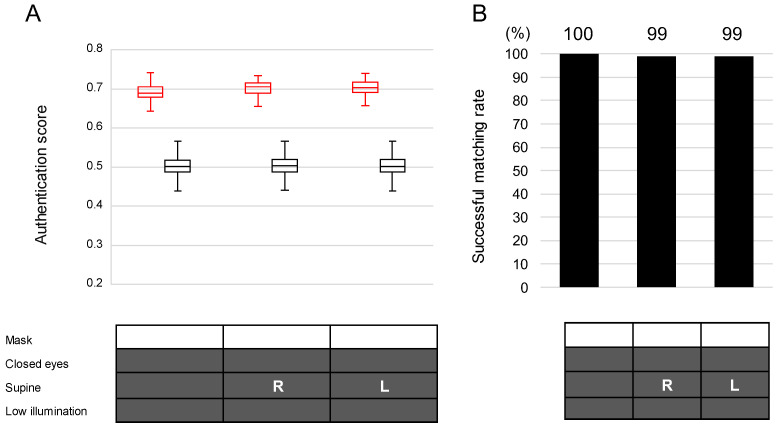
Authentication scores (**A**) and successful matching rates (**B**) in the nighttime condition. All images were taken under the low illumination condition. In the lower panel, solid boxes = conditions with mask, closed eyes, supine, and low illumination; R = right lateral position; L = left lateral position. In the upper panel in (**A**), red boxes = scores for correct patients, and black boxes = scores for wrong patients. The exact numbers are provided in the top row of the matching rate bar chart.

Under the nighttime sleeping condition, we successfully authenticated 99.3% (298/300) of the patients in either the left or right lateral position without a mask, in low light, and with closed eyes (Figure 5).

## 4. Discussion

In this study, we verified a facial recognition algorithm for hospital settings and obtained three essential findings. (1) The unmasked certification rate of 99.7% was significantly higher than the masked rate of 90.8%. For patients not wearing masks, the deep learning-based facial recognition system showed 99.7% accuracy even under adverse conditions (low illumination, eyes closed, supine position), excluding the normal condition. (2) Furthermore, the system showed 99.3% accuracy under nighttime sleeping conditions (supine position or lateral position, low illumination, closed eyes) without a mask. (3) For patients wearing a mask, the system showed 100% authentication accuracy if the patient was sitting with their eyes open. Previous studies on facial recognition have not been conducted among outpatients or inpatients in medical settings. This is the first study to perform facial recognition in different situations with combined conditions, e.g., the sitting position, supine position, under low lighting, or with eyelids closed, representing a hospital ward at night. 

The facial recognition system of this study learned ten million facial images of the general population regardless of race, age, or gender. This study focused on whether this system, which has already been implemented and disseminated in the general public, would work under different situations surrounding patients in a hospital. We set AS thresholds for masked and unmasked subjects because the minimum AS with a false acceptance rate (FAR) of 0% differs between masked and unmasked patients. Similar to security, zero wrong-patient acceptance is a prerequisite in the medical field, even if a mistake of excluding the correct person (false rejection rate (FRR) > 0%) disrupts on-site operations. The threshold value derived in this study was smaller for patients wearing masks (0.620) than for those without masks (0.642). These scores mean that judgments are made with a lower agreement with the reference photographs under the masked conditions, contributing to a lower percentage of correct responses. 

Among each adverse condition, the lowest AS was in the supine position. Soft skin changes its shape under gravity, and facial expressions differ between the supine and seated positions. This is why the AS was lower for images of patients in the supine position. The correct response rate remained good at 99%, even under adverse nighttime sleeping conditions, since the effects of eye closure and low illumination on AS, which add to the negative impact of posture, were smaller than those of wearing a mask. Conversely, even when wearing a mask, the AS remained relatively high in the sitting position (0.681 ± 0.020 and 0.688 ± 0.021), even with the addition of low illumination and closed eyes, resulting in reasonable response rates of 100% and 99%, respectively.

This study’s low illumination condition was 3–4 lux. The satisfactory results regarding the nighttime sleeping conditions in this study mean that we can use the system in a hospital room during actual nighttime hours. Recently, algorithms has improved and infrared 3D facial recognition has evolved to not reduce the authentication rate even in low illumination [27]. Facial recognition already had some advantages (i.e., the system does not need direct contact, works distantly, and does not rely on the patient’s response). Adding its performance during nighttime sleeping to these advantages improves the safety management of hospital rooms at night, when every hospital needs high operational efficiency due to the small number of nurses and the unique circumstance of not wanting to wake sleeping patients. According to the results of this study, we believe that this facial recognition system has the potential to be available even in hospitals, particularly for various conditions, such as patients without a mask or those sleeping at night. However, we need to note the rate of correct recognition depending on the underlying AI algorithm. 

The type of a camera that may be available in a hospital is important [9]. In this study, the photographs were taken with a device familiar to the general public, the iPad. Assuming medical treatment, facial recognition is performed at unspecified locations or times, so it is difficult to carry out using a fixed camera. It is necessary for medical professionals to be able to carry and operate the device [9]. Thus, the iPad is a suitable and useful tool for the complex situations of hospitals, and it is expected that the iPad can be implemented and disseminated in the medical field. 

This study is a pilot study evaluating how our facial recognition system could work in various settings in a hospital. The system has not yet been connected with electronic health records. This system is expected to be implemented in the hospital and contribute to the efficacy and safety of the patients by connecting with their electronic health records while protecting their privacy according to policy. In order to implement this system in hospitals, we confirmed the efficacy and safety of the facial recognition system in settled conditions in a hospital. However, we did not evaluate how our system performs under rapidly changing illumination or against complex backgrounds, particularly in an emergency setting. We need more data for the dissemination of this system under various settings in hospitals. 

There are several limitations to this study. First, the subjects of this study were cooperative patients. We did not evaluate the system’s performance for infants, patients with dementia, or unconscious people, for whom facial recognition would be especially effective because these people are difficult to authenticate using traditional methods such as call verification. Facial recognition accuracy for children is worse than that for adults, and the low quality of the recognition photo has been found to be the cause [28]. This is a subject to be addressed in the future. Research on a photographic technique that maintains the quality necessary to authenticate any subject will be required. Second, the subjects in this study were all Japanese and hospitalized, and most of them were around 70 years old. Therefore, we need to evaluate the data with the target of other races and ages for the dissemination of this system. Age or facial alternations due to medical conditions or treatments may affect the facial recognition score, and a future study is needed. Third, only one photographer took the authentication photographs. It has been reported that authentication performance is affected by the photographer’s experience [29]. Future studies should conduct authentication experiments with on-site staff, such as the nurses who will perform the authentications. Fourth, the effectiveness of facial recognition has not been evaluated in terms of cost, such as building a network backbone or personal information management. When this facial recognition system is used in multiple facilities, a cost-effectiveness evaluation will be necessary.

### Privacy Policy

The use of biometric data, such as those used for facial recognition, raises a new issue associated with personal information. Regarding the protection of personal information, the “face” is important information to identify an individual; thus, how to best handle these images is extremely important in order to protect personal information. [30,31]. The hospital follows the “Guideline for Safety Management of Medical Information Systems” (https://www.mhlw.go.jp/stf/shingi/0000516275_00006.html, accessed on 5 April 2024 Access date inserted.) specified by the Ministry of Health, Labor and Welfare. Companies are required by the Ministry of Economy, Trade and Industry to comply with the “Guidelines for Safety Management for Providers of Information Systems and Services that Handle Medical Information”. (https://www.meti.go.jp/policy/mono_info_service/healthcare/teikyoujigyousyagl.html, accessed on 5 April 2024), and Glory’s privacy policy is also presented on its website (https://www.glory.co.jp/info/privacy/, accessed on 5 April 2024).

In Japan, few hospitals have implemented facial recognition systems for patient identification. The implementation and dissemination of facial recognition systems may not be an easy task in other countries, as well as Japan, in the future. In Europe, they decided to strictly regulate AI with the AI act in 2024 according to “the proposal for a regulation of the European Parliament and of the Council on laying down harmonised rules on Artificial Intelligence (Artificial Intelligence Act) and amending certain Union Legislative Acts”. It stated, as follows, “To promote the uptake of human centric and trustworthy artificial intelligence (AI) while ensuring a high level of protection of health, safety, fundamental rights as enshrined in the Charter of fundamental rights of the European Union (the ‘Charter’), including democracy, the rule of law and environmental protection, against the harmful effects of AI systems in the Union, and to support innovation.” [32]. This ruling, the first in the world, is going to be carried out in 2026. This means that facial recognition systems have to be implemented with consideration of the trade-off relationship between personal information and patient safety.

## 5. Conclusions

Our study provides valuable insights into facial recognition technology’s efficacy in patient identification, especially in hospitals where accurate patient identification is crucial. The facial recognition system using deep learning for patient identification in a hospital setting showed promising results, proving its safety and acceptability.

## Figures and Tables

**Table 1:** Authentication score in each condition.

Mask	Eyes	Position	Illumination	Correct-Matching	Wrong-Matching
No	open	sitting	sufficient	0.785 ± 0.004	0.508 ± 0.025
No	closed	sitting	sufficient	0.767 ± 0.012	0.509 ± 0.025
No	open	supine	sufficient	0.710 ± 0.020	0.507 ± 0.024
No	closed	supine	sufficient	0.699 ± 0.021	0.507 ± 0.024
No	open	sitting	low	0.741 ± 0.012	0.503 ± 0.024
No	closed	sitting	low	0.731 ± 0.013	0.503 ± 0.024
No	open	supine	low	0.703 ± 0.018	0.503 ± 0.024
No	closed	supine	low	0.691 ± 0.019	0.502 ± 0.023
Yes	open	sitting	sufficient	0.714 ± 0.015	0.507 ± 0.022
Yes	closed	sitting	sufficient	0.688 ± 0.021	0.508 ± 0.022
Yes	open	supine	sufficient	0.659 ± 0.023	0.507 ± 0.021
Yes	closed	supine	sufficient	0.638 ± 0.027	0.506 ± 0.021
Yes	open	sitting	low	0.681 ± 0.020	0.504 ± 0.022
Yes	closed	sitting	low	0.664 ± 0.026	0.505 ± 0.021
Yes	open	supine	low	0.656 ± 0.022	0.503 ± 0.021
Yes	closed	supine	low	0.636 ± 0.027	0.502 ± 0.021
No	closed	supine R	low	0.701 ± 0.019	0.503 ± 0.024
No	closed	supine L	low	0.702 ± 0.019	0.503 ± 0.024

## Data Availability

The datasets generated during and/or analyzed during the current study are not publicly available but are available from the corresponding author upon reasonable request.
